# Role of Dopamine D2 Receptor in Stress-Induced Myelin Loss

**DOI:** 10.1038/s41598-017-10173-9

**Published:** 2017-09-14

**Authors:** Mi-Hyun Choi, Ji Eun Na, Ye Ran Yoon, Hyo Jin Lee, Sehyoun Yoon, Im Joo Rhyu, Ja-Hyun Baik

**Affiliations:** 1 Molecular Neurobiology Laboratory, Department of Life Sciences, Korea University, Seoul, 02841 Korea; 2 Department of Anatomy, College of Medicine, Korea University, Seoul, 02841 Korea

## Abstract

Dopaminergic systems play a major role in reward-related behavior and dysregulation of dopamine (DA) systems can cause several mental disorders, including depression. We previously reported that dopamine D2 receptor knockout (D2R^−/−^) mice display increased anxiety and depression-like behaviors upon chronic stress. Here, we observed that chronic stress caused myelin loss in wild-type (WT) mice, while the myelin level in D2R^−/−^ mice, which was already lower than that in WT mice, was not affected upon stress. Fewer mature oligodendrocytes (OLs) were observed in the corpus callosum of stressed WT mice, while in D2R^−/−^ mice, both the control and stressed group displayed a decrease in the number of mature OLs. We observed a decrease in the number of active β-catenin (ABC)-expressing and TCF4-expressing cells among OL lineage cells in the corpus callosum of stressed WT mice, while such regulation was not found in D2R^−/−^ mice. Administration of lithium normalized the behavioral impairments and myelin damage induced by chronic stress in WT mice, and restored the number of ABC-positive and TCF4-positive OLs, while such effect was not found in D2R^−/−^ mice. Together, our findings indicate that chronic stress induces myelin loss through the Wnt/β-catenin signaling pathway in association with DA signaling through D2R.

## Introduction

Dopamine (DA) regulates emotional and motivational behavior, and changes in dopaminergic neurotransmission have been found to modify behavioral responses to various environmental stimuli such as stress^1,2^. Stress is thought to be a key factor in the development of depression, as reported by numerous animal and human studies^3,4^. It has been suggested that the dopamine system is highly susceptible to stress and is altered by stressful stimuli; this alteration contributes to the pathophysiology of stress-induced depressive disorders^2,4–7^. Increasing evidence suggests that dopamine agonists are effective for treating depression in humans^8–10^.

The dopamine D2 receptor (D2R), which is one of the major DA receptor subtypes, is known to be critically involved in the pathology of depression and is associated with stress^11–13^. We have previously shown that anxiety-like and depression-like behaviors after chronic stress are more pronounced in D2R knockout mice (D2R^−/−^ mice) than in wild type (WT) mice^14^.

Depression is known to be associated with not only neuronal alteration but also glial dysfunction. In particular, it has been reported that patients with major depressive disorder display oligodendroglial abnormalities and alterations in oligodendrocyte (OL) structure, and that glial function can produce behavioral changes associated with mood regulation^15,16^. Transcriptional profiling analysis of patients with major depressive disorder revealed that changes in intercellular communication and signal transduction mechanisms that contribute to abnormalities in oligodendroglia and synaptic function may contribute to depressive disorder regulation^15^. Other studies have also shown significant reductions in the expression of myelin-related and OL-related genes in the brain of patients with depression^15,17–19^.

It has been reported that DA can affect myelin formation through OL development and function, in particular by D2Rs or D3Rs^20,21^, suggesting that a D2R agonist could increase the number of oligodendrocyte progenitor cells (OPCs). Notably, D2R agonism has been shown to provide significant protection of oligodendrocytes against oxidative injury^22^.

The current study addressed whether DA signals via D2R regulate myelination in stress-induced depression by examining myelination changes in response to chronic stress in WT and D2R^−/−^ mice. We found that stress induced a loss of myelination in WT mice which was protected with treatment of the antidepressant, lithium. D2R^−/−^ mice showed alterations in this stress-induced myelin loss; however, they were not responsive to lithium treatment. Our results show that the D2R has a key role in altering myelination, which can be associated with pathology of DA-related neuropsychiatric diseases.

## Results

### Chronic stress-induced myelin loss in WT and D2R^−/−^ mice

**Figure 1:**
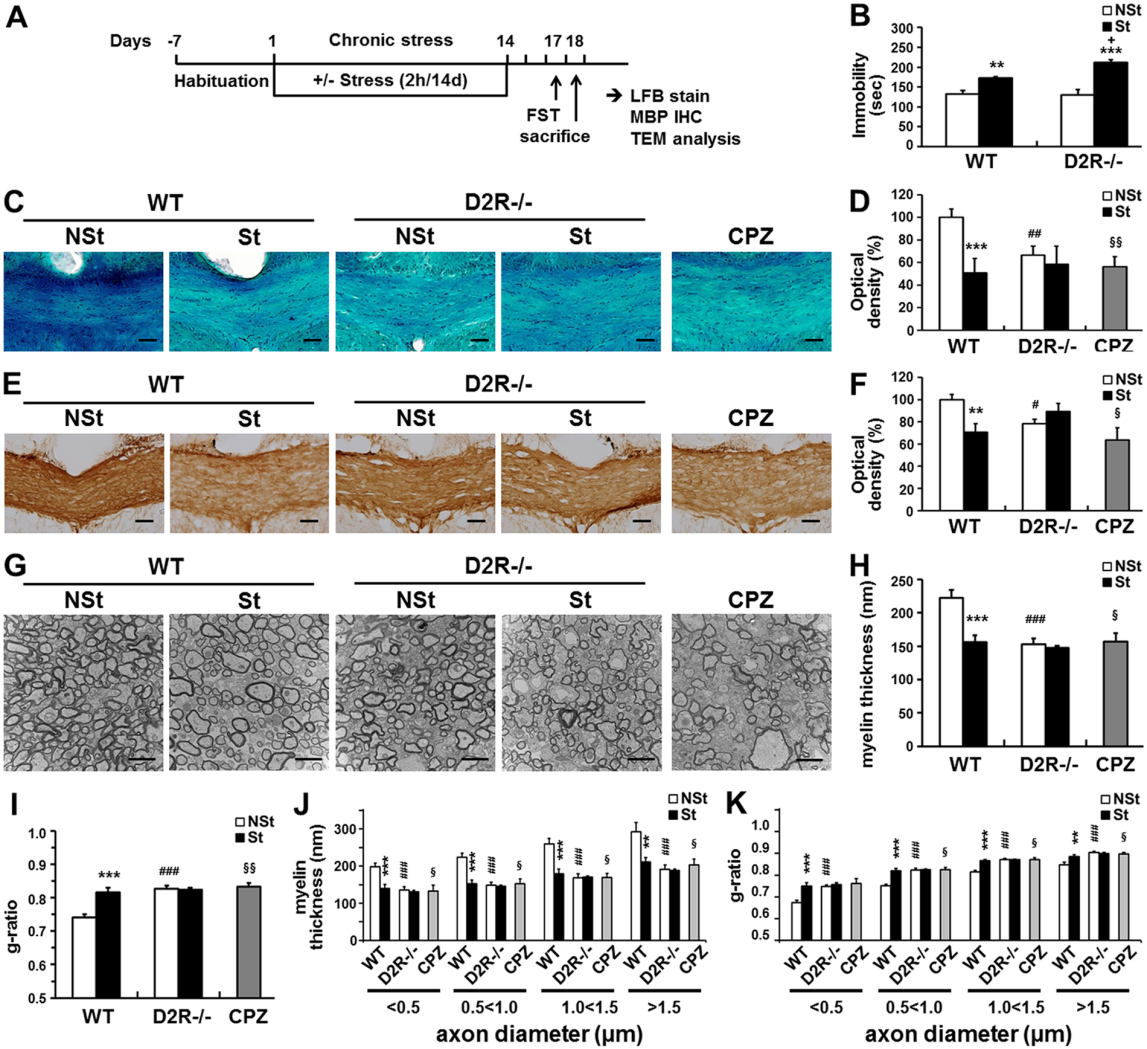
Effects of chronic stress on myelination in WT and D2R^−/−^ mice. (**A**) Schematic illustration of the chronic immobilization stress model. For chronic stress, WT and D2R^−/−^ mice were immobilized for 2 h once daily for 14 days. Control groups of mice were left undisturbed. (**B**) Immobilization time after chronic stress. Mice were subjected to the forced swim test after exposure to chronic restraint stress (St). Non-stressed (NSt) animals were examined as a control (WT-NSt n = 11, WT-St n = 7, D2R^−/−^-NSt n = 8, D2R-St n = 8). (**C**–**F**) Visualization of myelin by luxol fast blue (LFB) staining (**C**) and myelin basic protein (MBP) immunohistochemistry (**E**). WT mice following 5 weeks of cuprizone feeding (CPZ) were examined as the myelin loss control. (**D**,**F**) Quantitative analysis of LFB staining and MBP immunohistochemistry by integrated optical densitometry within the middle corpus callosum (region from bregma 0.02 mm to −0.82 mm). Scale bars: 50 μm (**D**, WT-NSt n = 6, WT-St n = 5, D2R^−/−^-NSt n = 6, D2R-St n = 5, CPZ n = 6; (**F**) WT-NSt n = 7, WT-St n = 6, D2R^−/–^-NSt n = 6, D2R-St n = 5, CPZ = 6) (**G**–**K**), Transmission electron micrographs of transverse sections at the middle corpus callosum (bregma −0.46 mm, mid-sagittal 0 mm) (n = 5, scale bars represent 2 μm). Approximately 1500 axons from 5 mice were investigated in each group. Average myelin thickness (**H**) and g-ratio (**I**) in the middle corpus callosum were measured and grouped by axon diameter (**J**,**K**) (n = 5 mice for each group). White bars indicate non-stressed animals, black bars indicate stressed animals and gray bars indicate cuprizone-fed animals. Data are presented as mean ± SEM. **P < 0.01, ***P < 0.001 versus NSt mice; ^#^P < 0.05, ^##^P < 0.01, ^###^P < 0.001 versus WT-NSt mice; ^+^P < 0.01 versus genotype (Two-way ANOVA post hoc test). ^§^P < 0.05, ^§§^P < 0.01 versus WT-NSt mice (Two-tailed Student’s t-test).

WT and D2R^−/−^ mice assigned to “stressed” groups were subjected to restraint stress by immobilization in a restrainer for 2 h daily for 14 d, as described previously^14^ (Fig. 1A). Control groups of mice were left undisturbed. After stress exposure, WT and D2R^−/−^ mice subjected to chronic stress were evaluated in the forced swim test. In this test, immobility time is considered an indicator of a depressive state. The stress-induced increase in immobility time in D2R^−/−^ mice was significantly longer than that of WT mice (stress × genotype interaction: F_1,30_ = 5.43, P = 0.027; Fig. 1B). This finding is in accordance with previous data showing increased levels of depressive behaviors in D2R^−/−^ compared to WT animals following stress^14^.

We determined whether chronic stress affects myelination in WT and D2R^−/−^ mice. Luxol fast blue (LFB) myelin staining was used for measuring the extent of myelin loss^23^. As a myelin loss control, we used the copper chelator cuprizone (CPZ), which can induce severe demyelination with oligodendrocyte damage in animals after 5 to 6 weeks of CPZ feeding^24,25^ (Fig. 1C). We observed severe myelin loss of the corpus callosum of stressed WT mice (WT-St) compared to non-stressed control WT mice (WT-NSt) (Fig. 1C,D). By analyzing the density of LFB staining, the white matter of the corpus callosum spanning from bregma 1.10 mm to −2.30 mm was evaluated. We found that most of the corpus callosum between 0.14 mm and −0.82 mm was significantly demyelinated in the WT-St group mice as compared to the control group. This loss of white matter was comparable with the cuprizone (CPZ)-fed (for 5 weeks) animals, where the LFB staining intensity was decreased up to 55% in the corpus callosum. Interestingly, a low level of LFB staining density was already observed in non-stressed D2R^−/−^ mice (D2R^−/−^-NSt) (51% versus WT-NSt group) and stressed D2R^−/−^ mice (D2R^−/−^-St) group displayed a similar level of LFB staining density (Fig. 1C,D, stress × genotype interaction: F_1,18_ = 5.65, P = 0.029). In parallel, we have also analyzed the representative myelin-specific protein, Myelin Basic Protein (MBP)^26^ in the corpus callosum area by immunohistochemistry and observed a significant decrease of MBP expression in the WT-St mice compared to the WT-NSt control group (71% of WT-NSt group). In D2R^−/−^ mice, the non-stressed control group showed a low level of MBP which was not affected by stress subjection (78% of control WT mice, stress × genotype interaction: F_1,20_ = 11.33, P = 0.003; Fig. 1E,F). These findings suggest that myelin is decreased by stress in WT mice while the basal myelin level is low in D2R^−/−^ mice, and that there is no change of myelin status by chronic stress in these mice.

Ultrastructural analysis of myelinated axons in the corpus callosum by electron microscopy revealed a significant decrease in myelin thickness of axons within different diameter scales (223 ± 11 nm in WT-NSt control, n = 5 and 156 ± 10 nm, in WT-St, n = 5) and an increase in the g-ratio (0.741 ± 0.010 in WT-NSt, n = 5 and 0.816 ± 0.013, WT-St, n = 5, g-ratio: axon diameter/outer myelin sheath diameter) in the WT-St group compared to the WT-NSt group (Fig. 1G–K). Approximately 1500 axons from 5 mice were investigated in each group. In the corpus callosum of D2R^−/−^-NSt control mice; however, basal myelin thickness was already low (153 ± 8 nm, n = 5, 69% of control WT mice, myelin thickness: stress × genotype interaction: F_1,16_ = 11.83, P = 0.003; Fig. 1H) and the g-ratio was increased as compared to WT control mice (0.827 ± 0.009, n = 5, 112% of control WT mice, stress × genotype interaction: F_1,16_ = 16.23, P = 0.001; Fig. 1I). A similar pattern of myelin level was observed in D2R^−/−^-St mice (myelin thickness: 148 ± 3 nm, n = 5, g-ratio: 0.823 ± 0.007, n = 5). This myelin level in D2R^−/−^ mice was comparable to that of CPZ-fed animals (myelin thickness: 157 ± 13 nm, n = 5; g-ratio, 0.833 ± 0.010, n = 5). These myelin thickness and g-ratio changes were observed across the different axon diameter scales (stress × genotype interaction: myelin thickness, category < 0.5, F_1,16_ = 8.8, P = 0.009; category 0.5 < 1.0, F_1,16_ = 15.02, P = 0.001; category 1.0 < 1.5, F_1,16_ = 13.46, P = 0.002; category > 1.5, F_1,16_ = 6.74, P = 0.02; g-ratio, category < 0.5, F_1,16_ = 8.75, P = 0.009; category 0.5 < 1.0, F_1,16_ = 18.92, P = 0.0005; category 1.0 < 1.5, F_1,16_ = 22.13, P = 0.0002; category > 1.5, F_1,16_ = 7.07, P = 0.017; paired, two-tailed Student’s t-test: myelin thickness, category < 0.5, P = 0.0048; category 0.5 < 1.0, P = 0.0291; category 1.0 < 1.5, P = 0.0168; category > 1.5, P = 0.046; for WT non-stress versus CPZ: g-ratio, category < 0.5, P = 0.051; category 0.5 < 1.0, P = 0.0151; category 1.0 < 1.5, P = 0.0142; category > 1.5, P = 0.002, Fig. 1J,K). These results strongly indicate that chronic stress can cause severe myelin loss in adult WT mice and that the absence of D2R results in loss of myelin and similar level of myelin loss was observed in both D2R^−/−^-NSt and D2R^−/−^-St mice.

### Stress-induced myelin loss involves a decrease in mature oligodendrocytes in WT mice

**Figure 2:**
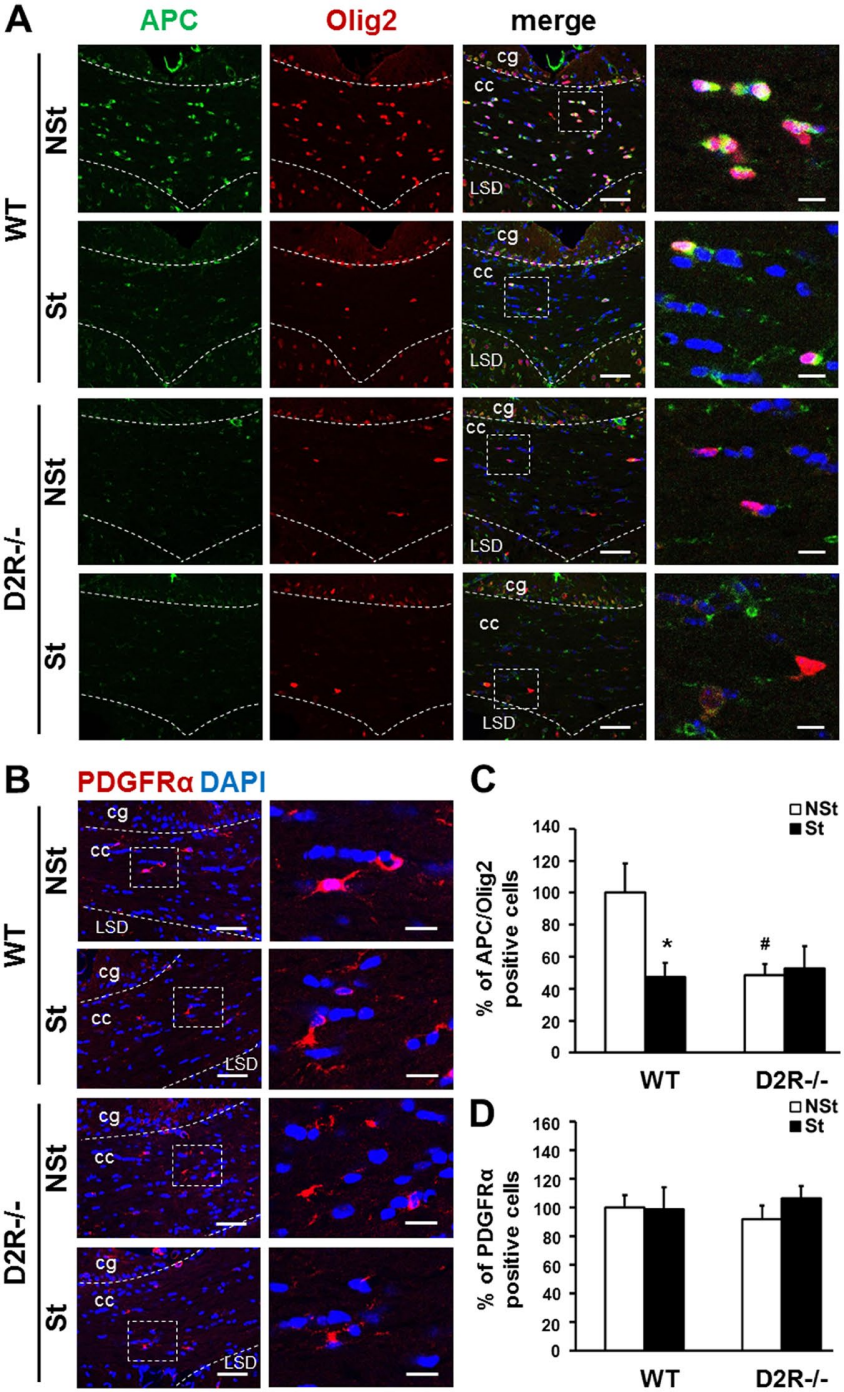
Quantitative analysis of mature oligodendrocytes in stress-imposed WT and D2R^−/−^ mice. (**A**,**B**) Identification of mature oligodendrocytes and oligodendrocyte precursor cells after chronic stress. Immunolabeling performed using antibodies to mature oligodendrocyte marker APC (CC1 clone), oligodendrocyte lineage marker Olig2, and premature oligodendrocyte marker PDGFRα. Representative low and high magnification immunofluorescence images of APC(green)/Olig2(red) (**A**) and PDGFRα (red)- positive cells (**B**) in the middle corpus callosum of stress-imposed WT and D2R^−/−^ mice (St). Non-stressed (NSt) animals were examined as a control. White dashed line indicates corpus callosum region and white dashed box shows magnified image presented at far right panel. Scale bars: 50 μm in low magnification images and 10 μm in high magnification images. cc: corpus callosum, cg: cingulate gyrus, LSD: lateral septal nucleus. (**C**,**D**) The percentage of APC/Olig2 and PDGFRα positive cells in middle corpus callosum for non-stressed and stressed animals in WT and D2R^−/−^ mice (**C**) WT-NSt n = 8, WT-St n = 7, D2R^−/−^-NSt n = 7, D2R-St n = 6; (**D**) n = 8 mice for each group). White bars indicate non-stressed animals, black bars indicate stressed animals. Data are presented as mean ± SEM. *P < 0.05 versus NSt mice; ^#^P < 0.05 versus WT mice (Two-way ANOVA post hoc test).

To examine in more detail the altered myelination in stressed WT and D2R^−/−^ mice, we identified OL cell populations in the corpus callosum of each group and determined the number of oligodendrocyte precursor cells (OPCs) and mature OLs among all the OL lineage cells. PDGFRα (platelet-derived growth factor receptor, alpha polypeptide) is a known marker of oligodendrocyte precursors^27^, and it has been shown that the *adenomatous polyposis coli* (APC) protein is a marker for mature OLs^28^. Oligodendrocyte transcription factor (Olig2) is a basic helix-loop-helix (bHLH) transcription factor encoded by the *Olig2* gene and Olig2 labels OPCs and mature OLs, and is thus indicative of all OL lineage cells^29^. The number of OPCs (PDGFRα+), APC-positive cells (APC+, representing mature OLs), and Olig2-positive cells (Olig2+, representing all OL lineage cells) were enumerated in control and stressed animals. By double immunofluorescence analysis, we found that in the corpus callosum of WT-St mice, Olig2+ and APC+ cells were decreased and APC+/Olig2+ cells were decreased by 47% of the WT-NSt control group (Fig. 2A,C). In the corpus callosum of D2R^−/−^ mice, both D2R^−/−^-NSt and D2R^−/−^-St groups of mice displayed a significant decrease of Olig2+ and APC+ cells, and APC+/Olig2+ cells by 48–53% of WT control mice (D2R^−/−^-NSt mice, 48% of WT-NSt mice; D2R^−/−^-St mice, 53% of WT-NSt mice, stress × genotype interaction: F_1,24_ = 4.55, P = 0.043, Fig. 2A,C). In contrast, the number of PDGFRα+ cells was comparable across the corpus callosum of both WT and D2R^−/−^ mice, and was not changed by stress (stress × genotype interaction: F_1,28_ = 0.54, P = 0.47, Fig. 2B,D). These results suggest that chronic stress-induced myelin loss involves a decrease in the number of mature OLs in WT mice and in D2R^−/−^ mice, the latter which already had decreased number of mature OLs and similar level of OLs number is observed in D2R^−/−^-St mice.

### Wnt/β-catenin-TCF signaling is associated with stress-induced myelin loss

Further, we examined the mechanism by which stress-induced myelin loss is mediated and determine why a low level of myelin is observed in D2R^−/−^ mice despite similar level of OPC cells as WT mice.

Recent studies have shown that the Wnt/β-catenin-TCF/LEF signaling pathway is critical in the regulation of oligodendrogenesis, OL differentiation, and myelination^29–35^, although the exact role of Wnt signaling in the regulation of myelination appears contradictory. We recently found that the D2R can interact with the Wnt protein, suggesting that Wnt may play an important role in dopaminergic neuronal development through the ERK pathway^36,37^. Based on these findings, we hypothesized that Wnt/β-catenin-TCF/LEF signaling might be involved in the stress-induced myelin loss associated with D2R.

**Figure 3:**
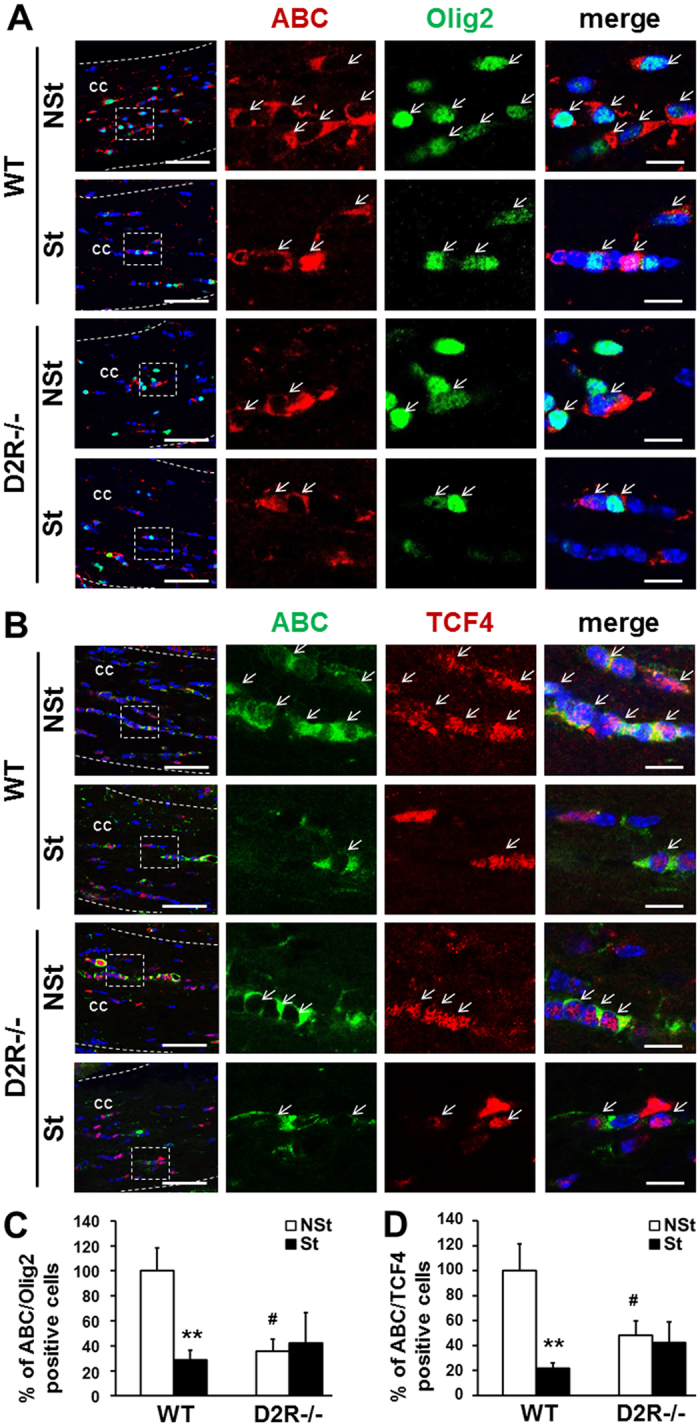
Regulation of Wnt signaling in the corpus callosum of stress-imposed WT and D2R^−/−^ mice. (**A**,**B**) Wnt signaling inactivation after chronic stress in WT and D2R^−/−^ mice. Immunolabeling performed using antibodies to active β-catenin (ABC), Olig2 and TCF4. Representative low and high magnification immunofluorescence images of ABC (red)/Olig2 (green) (**A**), ABC (green)/TCF4 (red) (**B**), double labeled cells were observed in the middle corpus callosum of stress-imposed WT and D2R^−/−^ mice (St). Non-stressed (NSt) animals were examined as a control. White arrows indicate double-labeled cells. White dashed line indicates corpus callosum region and white dashed box shows magnified image presented at far right panel. Scale bars: 50 μm in low magnification images and 10 μm in high magnification images. cc: corpus callosum. (**C**,**D**) Percentage of ABC/Olig2 and ABC/TCF4 positive cells in middle corpus callosum for non-stressed and stressed animals in WT and D2R^−/−^ mice (**C**) WT-NSt n = 5, WT-St n = 5, D2R^−/−^-NSt n = 4, D2R-St n = 4; (**D**) WT-NSt n = 7, WT-St n = 7, D2R^−/−^-NSt n = 6, D2R-St n = 6). White bars indicate non-stressed animal, black bars indicate stressed animals. Data are presented as mean ± SEM. **P < 0.01, ***P < 0.001 versus NSt mice; ^#^P < 0.05, ^##^P < 0.001 versus WT mice (Two-way ANOVA post hoc test).

To determine whether the β-catenin/TCF4 signaling is involved in stress-induced myelin loss, we first analyzed active β-catenin (ABC) expression in Olig2+ cells in the corpus callosum of control and stressed mice from WT and D2R^−/−^ mice. We observed a significant decrease in the number of ABC expressing-cells in OL lineage cells, with Olig2+ cells (ABC+/Olig2+) of WT-St mice reduced by 30% of that of non-stressed control group (29 ± 8%, n = 5 of WT-NSt mice, n = 5, Fig. 3A,C). In the D2R^−/−^ mice, control mice showed a significant decrease in the number of ABC+/Olig2+ cells (36 ± 9% of the WT-Nst control group, n = 4); however, the number of ABC/Olig2 double-labeled cells was not changed by stress in D2R^−/−^ mice (42 ± 24% of WT-NSt mice, n = 4; stress × genotype interaction: F_1,26_ = 7.58, P = 0.0106, Fig. 3A,C).

Double immunofluorescence analysis with antibodies against ABC and TCF4 revealed that the number of ABC+/TCF4+ double-labeled cells were significantly decreased in WT-St mice (22 ± 5% of WT-NSt mice, n = 7, Fig. 3B,D). Non-stressed D2R mice, which were previously shown to have a reduced number of ABC+/TCF4+, showed a reduction of ~48% of control WT mice (48 ± 11%, n = 6); this change was nominally affected by stress in D2R^−/−^ mice (42 ± 17% of control WT-NSt mice, n = 6, Fig. 3B,D). These results suggest that the β-catenin/TCF4 signaling is an important mediator in stress-induced myelin loss and that the absence of D2R blunts this regulation.

### Effect of lithium on stress-induced myelin loss

Since the chronic stress-induced depressive state evoked such significant myelin loss, we next investigated the ability of an antidepressant to modulate myelination.

**Figure 4:**
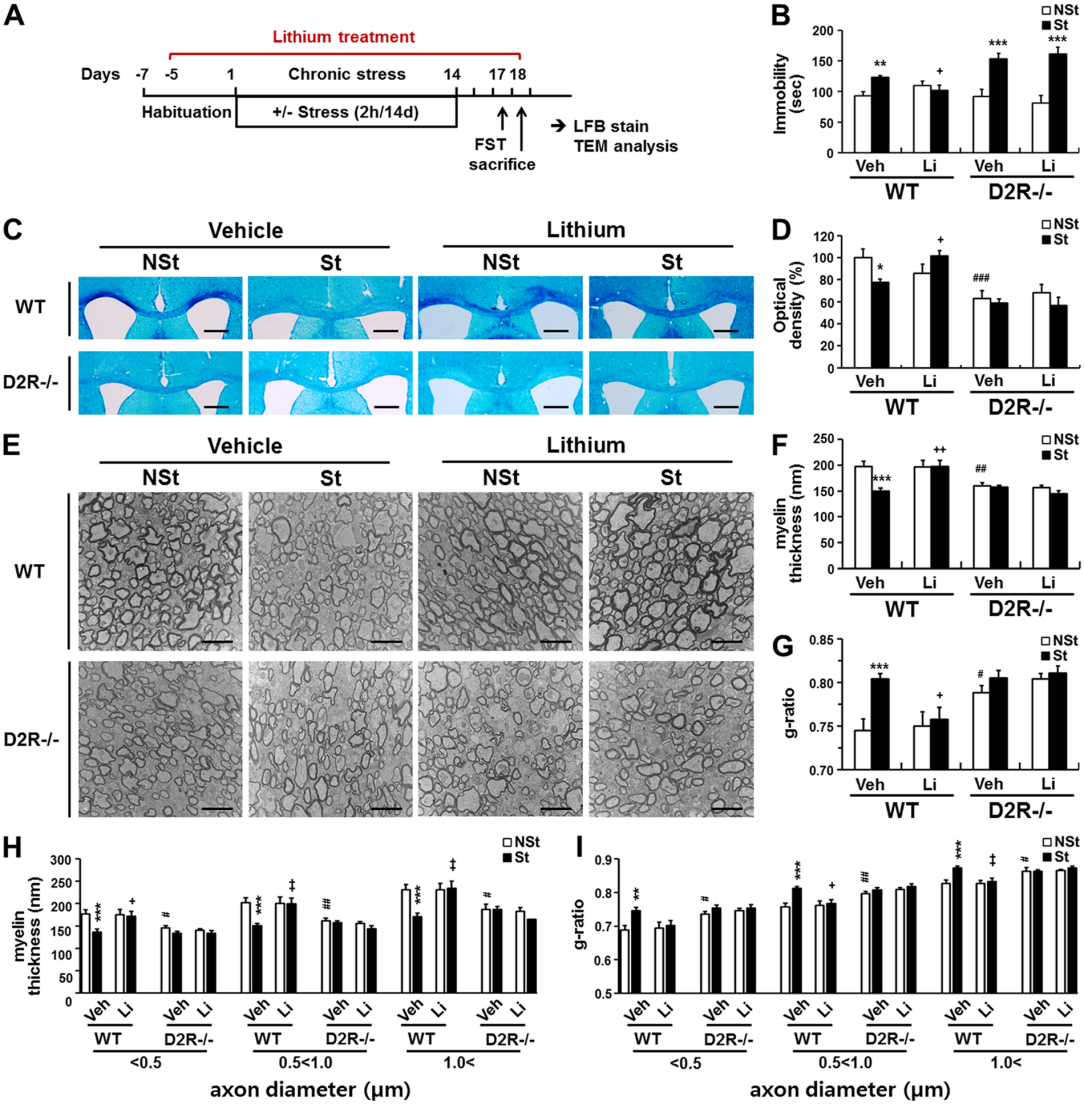
Effects of lithium treatment to stress-induced behavioral impairment and myelin loss. (**A**) Schematic illustration of the chronic immobilization stress and lithium treatment model. (**B**) Antidepressant-like effects of lithium (Li) after 2 weeks of chronic immobilization stress in the forced swimming test. Non-treated animals (Veh) were examined as a control for lithium treatment (WT-NSt Veh n = 16, WT-St Veh n = 15, WT-NSt Li n = 13, WT-St Li n = 15, D2R^−/−^-NSt Veh n = 13, D2R^−/−^-St Veh n = 12, D2R^−/−^-NSt Li n = 13, D2R^−/−^-St Li n = 10). (**C**) Restored myelin levels by lithium treatment. After behavioral test, myelination was visualized by LFB staining. Scale bar: 500 μm. (**D**) Quantitative analysis of LFB staining by optical density in middle corpus callosum (five sections per mouse, region from bregma 0.02 mm to −0.82 mm, WT-NSt Veh n = 7, WT-St Veh n = 8, WT-NSt Li n = 7, WT-St Li n = 7, D2R^−/−^-NSt Veh n = 7, D2R^−/−^-St Veh n = 8, D2R^−/−^-NSt Li n = 7, D2R^−/−^-St Li n = 7). (**E**–**I**) Transmission electron micrographs of transverse sections at the middle corpus callosum (bregma −0.46 mm, mid-sagittal 0 mm) from WT and D2R^−/−^ mice subjected to chronic stress with or without lithium treatment. Average of myelin thickness (**F**) and g-ratio (**G**) in the middle corpus callosum were measured and grouped by axon diameter (**H**,**I**). Approximately 300 axons were investigated in each mouse. Scale bar: 2 μm (WT-NSt Veh n = 7, WT-St Veh n = 7, WT-NSt Li n = 6, WT-St Li n = 6, D2R^−/−^-NSt Veh n = 4, D2R^−/−^-St Veh n = 6, D2R^−/−^-NSt Li n = 4, D2R^−/−^-St Li n = 5). Data are presented as mean ± SEM. White bars indicate non-stressed animals and black bars indicate stressed animals. Veh: vehicle, Li: lithium, NSt: non-stressed mice, St: stress-imposed mice. *P < 0.05, **P < 0.01, ***P < 0.001 versus NSt mice; ^#^P < 0.05, ^##^P < 0.01, ^###^P < 0.001 versus genotype. ^+^P < 0.05, ^++^P < 0.01, ^+++^P < 0.001 versus lithium treatment (Two-way ANOVA post hoc test).

Lithium, a glycogen synthase kinase 3β (GSK3β) inhibitor, is widely used in the treatment of mood disorders, managing both bipolar disorder and unipolar depression^38–40^. WT and D2R^−/−^ mice, which were subjected to chronic stress with or without lithium treatment, were evaluated in the forced swim test (FST), and further examined for myelination changes (Fig. 4A). Lithium treatment significantly lowered the immobility time in WT mice to levels almost near those of non-stressed control WT mice (93 ± 7 sec, n = 16 in control WT-NSt mice; 123 ± 3 sec, n = 15 in WT-St mice; 102 ± 8 sec, n = 15, in lithium-treated WT-St mice, Fig. 4B). However, lithium did not have any effect on immobility time in D2R^−/−^ mice (92 ± 12 sec, n = 13 in control D2R^−/−^-NSt mice; 154 ± 9 sec, n = 12 in D2R^−/−^-St mice; 161 ± 12 sec, n = 10, in lithium treated D2R^−/−^-St mice, Fig. 4B). Therefore, as previously observed, D2R^−/−^ mice are susceptible to the chronic stress-induced depressive state (stress × genotype interaction: F_1,52_ = 4.12, P = 0.048). Furthermore lithium treatment was not effective in D2R^−/−^ mice (stress × Li interaction in WT mice: F_1,55_ = 8.36, P = 0.0055; Fig. 4B).

Lithium treatment effectively restored the myelination in stressed WT mice, as revealed by LFB staining (Fig. 4C,D). We evaluated the level of myelin by LFB staining within the middle corpus callosum (five sections, region from bregma 0.14 mm to −0.82 mm, 0.19 mm intervals that showed severe myelin loss with LFB and MBP staining, Fig. 1C–F). Lithium treatment increased the intensity of LFB staining in stressed WT mice, in agreement with the behavioral and stress-induced myelin loss results (WT-St mice, n = 8, 78 ± 3% of control WT-NSt mice, n = 7; 102 ± 5%, n = 7 in lithium treated WT-St mice, Fig. 4C,D). However, lithium did not show any effect in D2R^−/−^ mice (63 ± 7% of control WT-NSt mice in D2R^−/−^-NSt mice, n = 7; 64 ± 3%, n = 8 in D2R^−/−^-St and 56 ± 8%, n = 7, in lithium-treated D2R^−/−^-St mice, stress × genotype interaction: F_1,29_ = 7.74, P = 0.009, stress × Li interaction in WT mice: F_1,25_ = 9.21, P = 0.006, Fig. 4C,D).

Electron microscopic analyses of the corpus callosum of WT and D2R^−/−^ mice were performed independently from the experimental group illustrated in Fig. 1. In WT mice, lithium treatment recovered the myelin thickness and g-ratio in stressed animals (myelin thickness, 198 ± 10 nm, n = 7 in control WT-NSt; 150 ± 6 nm, n = 7 in WT-St; 197 ± 12 nm, n = 6, in lithium-treated WT-St mice; g-ratio, 0.745 ± 0.013, n = 7 in control WT-NSt; 0.804 ± 0.006, n = 7 in WT-St; 0.758 ± 0.014, n = 6 in lithium-treated WT-St mice, Fig. 4E–G). D2R^−/−^ mice had thinner myelin sheaths (160 ± 6 nm, n = 4) and higher g-ratio (0.788 ± 0.008, n = 4) compared to WT mice, and myelin thickness and g-ratio was similar in D2R^−/−^-St mice (157 ± 4 nm and 0.805 ± 0.009, respectively, n = 6, Fig. 4E–G) as previously observed in Fig. 1. Lithium treatment in D2R^−/−^-St mice did not change myelin thickness and g-ratio (144 ± 7 nm and 0.811 ± 0.008, n = 5). These effects were observed across different sizes of axons (myelin thickness, stress × Li interaction in WT mice: F_1,22_ = 5.75, P = 0.025, stress × genotype interaction: F_1,20_ = 9.44, P = 0.006; category 0.5 < 1.0, stress × Li interaction in WT mice: F_1,22_ = 5.48, P = 0.029, stress × genotype interaction: F_1,20_ = 9.36, P = 0.006, category 1.0 < , stress × Li interaction in WT mice: F_1,22_ = 6.14, P = 0.021, stress × genotype interaction: F_1,20_ = 9.12, P = 0.007; g-ratio, category 0.5 < 1.0, stress × Li interaction in WT mice: F_1,22_ = 5.84, P = 0.024, stress × genotype interaction: F_1,20_ = 7.06, P = 0.015, category 1.0 < , stress × Li interaction in WT mice: F_1,22_ = 5.07, P = 0.035, stress × genotype interaction: F_1,20_ = 8.35, P = 0.009, Fig. 4H,I). Together, these findings suggested that lithium treatment promoted the functional recovery of stress-induced behavioral abnormality and rescued myelin damage, and this recovery was not seen in D2R^−/−^ mice.

### Effect of lithium on β-catenin/TCF4 signaling pathway

**Figure 5:**
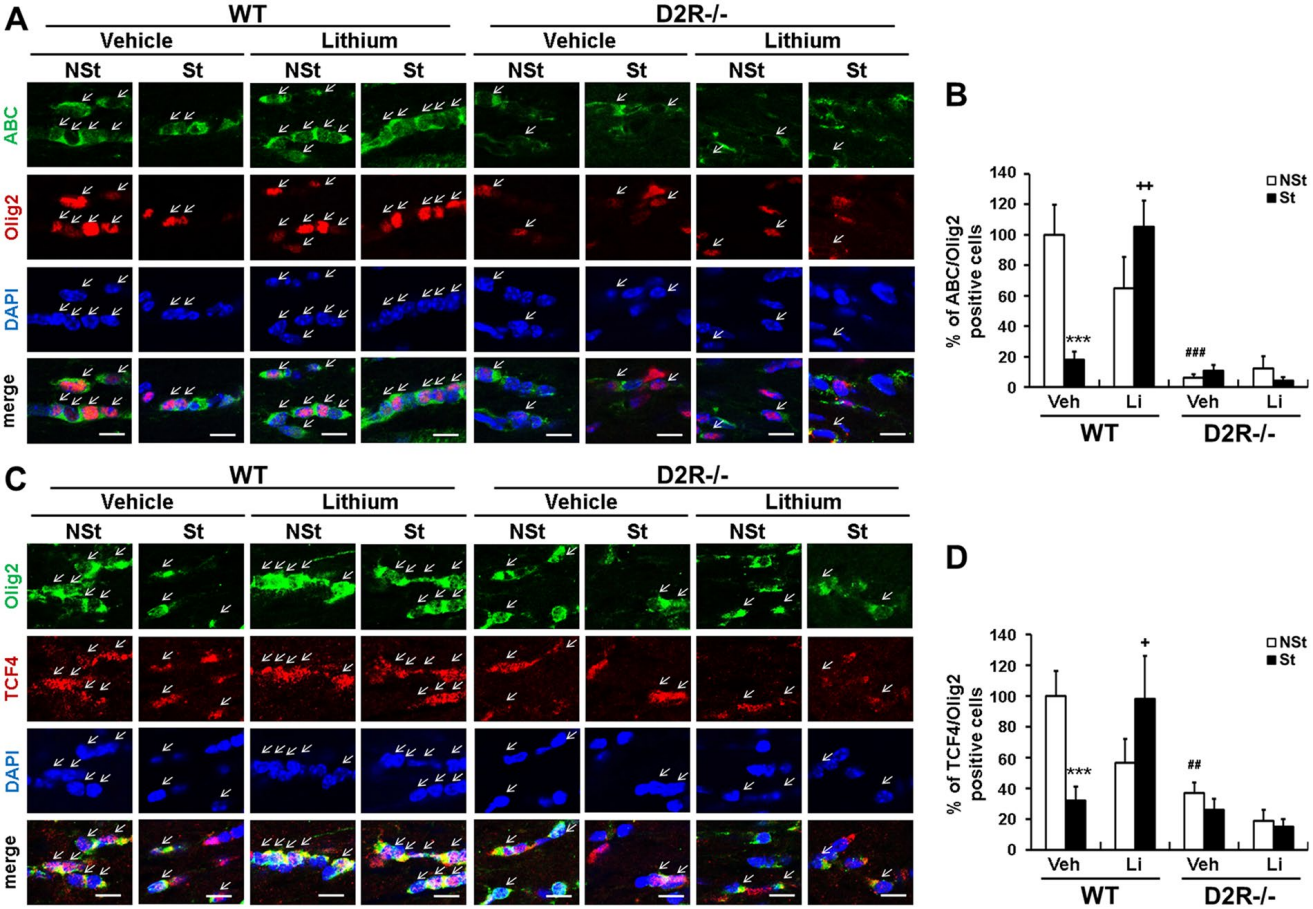
Effects of lithium treatment for Wnt signaling components expression in stress-imposed WT and D2R^−/−^ mice. (**A**,**C**) Restoration of Wnt signaling by lithium treatment. Immunolabeling performed using antibodies to active β-catenin (ABC), Olig2 and TCF4. High magnification immunofluorescence images of ABC (green)/Olig2 (red) (**A**) and TCF4 (red)/Olig2 (green) (**C**) positive cells in the middle corpus callosum (bregma −0.46 mm) in stress-imposed WT and D2R^−/−^ mice (St) with/without lithium treatment. Scale bars: 20 μm. (**B**,**D**) Percentage of ABC/Olig2 and TCF4/ Olig2 double-positive cells in the middle corpus callosum of WT and D2R^−/−^ mice (**B**) WT-NSt Veh n = 5, WT-St Veh n = 5, WT-NSt Li n = 4, WT-St Li n = 6, D2R^−/−^-NSt Veh n = 4, D2R^−/−^-St Veh n = 6, D2R^−/−^-NSt Li n = 3, D2R^−/−^-St Li n = 4; (**D**) WT-NSt Veh n = 7, WT-St Veh n = 7, WT-NSt Li n = 5, WT-St Li n = 6, D2R^−/−^-NSt Veh n = 6, D2R^−/−^-St Veh n = 6, D2R^−/−^-NSt Li n = 4, D2R^−/−^-St Li n = 4). White bars indicate non-stressed animals, black bars indicate stressed animals. Data are presented as mean ± SEM. Veh: vehicle, Li: lithium, NSt: non-stressed mice, St: stress-imposed mice. ***P < 0.001 versus NSt mice; ^##^P < 0.01, ^###^P < 0.001 versus WT mice; ^+^P < 0.05, ^++^P < 0.01 versus lithium treatment (Two-way ANOVA post hoc test).

We then addressed whether lithium can alleviate stress-induced myelin loss through Wnt signaling. Double-immunostaining for ABC+/Olig2+ cells revealed that lithium treatment restored the number of ABC-positive OLs in WT-St mice (18 ± 6%, n = 5 in WT-St and 105 ± 17%, n = 6 in lithium treated WT-St mice, Fig. 5A,B). However, the number of ABC+/Olig2+ cells was not changed by lithium treatment in D2R^−/−^-St mice, which had a low number of ABC+/Olig2+ cells (6 ± 2% of control WT-NSt, n = 4 in control D2R^−/−^-NSt, 11 ± 4%, n = 6 in stressed D2R^−/−^-St and 4 ± 2%, n = 4 in lithium treated D2R^−/−^-St mice, Fig. 5A,B). Therefore, lithium treatment induced recovery of ABC+/Olig2+ cells in stressed WT mice but not in D2R^−/−^ mice (stress × Li interaction in WT mice: F_1,17_ = 12.85, P = 0.002; stress × genotype interaction: F_1,16_ = 16.75, P = 0.001, Fig. 5A,B).

Simultaneously, double immunostaining for TCF4+/Olig2+ revealed a similar result, showing a decrease in the number of TCF4-positive OLs by chronic stress in WT mice (32 ± 9% of control WT mice, n = 7, Fig. 5C,D) and a restoration in the number of TCF4+/Olig2+ cells in lithium-treated WT-St mice (98 ± 28% of control WT mice, n = 6, Fig. 5C,D). As shown in Fig. 5C and D, the number of TCF4-positive OLs was lower in D2R^−/−^ mice than in WT mice (37 ± 7%, n = 6) and not changed by chronic stress (26 ± 7%, n = 6); however, a rather slight decrease was observed with lithium treatment in D2R^−/−^-St mice (15 ± 5%, n = 4, Fig. 5C,D). Thus, we conclude that lithium treatment normalized chronic stress-induced Wnt signaling inactivation, and D2R is required for this regulation (stress × Li interaction in WT mice: F_1,21_ = 9.01, P = 0.007; stress × genotype interaction: F_1,22_ = 6.72, P = 0.017, Fig. 5C,D).

### Effect of D2R activation on stress-induced myelin loss

**Figure 6:**
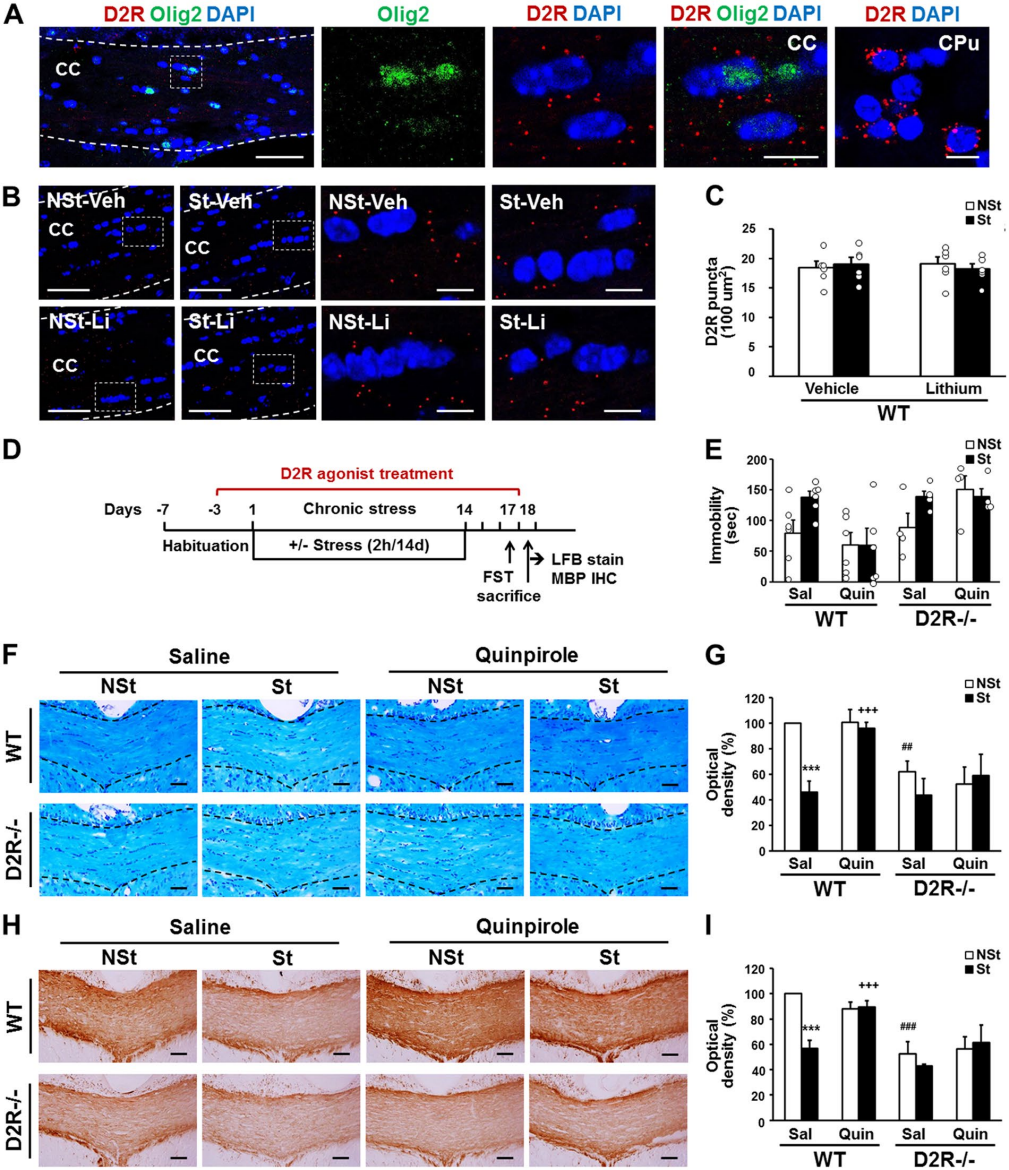
Effects of quinpirole treatment on stress-induced depressive behavior and myelin loss. (**A**) D2R mRNA (red) and Olig2 protein (green) expression in adult mouse corpus callosum (bregma -0.46 mm). D2R expression labelled by FISH in CPu is shown as positive control. (**B**) D2R mRNA expression in corpus callosum of non-treated (Veh) and lithium treated (Li) stress-imposed (St) or non-stressed control (NSt) mice group. White dashed line indicates corpus callosum region and white dashed box indicates magnified image. CPu: caudate putamen, CC: corpus callosum. Scale bars: 50 μm in low magnification images and 10 μm in high magnification images. (**C**) D2R mRNA puncta count per 100 um^2^ area in the middle corpus callosum region from bregma −0.22 mm, −0.46 mm and −0.70 mm (3 sections per mouse) from WT mice subjected to chronic stress with or without lithium treatment. Open circle denotes raw data for each group. (**D**) Schematic illustration of the chronic immobilization stress and quinpirole treatment experiment. (**E**) Effects of saline control (Sal) or quinpirole treatment (Quin, 5 mg/kg, daily i.p. injected one hour before restraint stress) during 2 weeks of chronic immobilization stress on the forced swimming test. (WT n = 6, D2R^−/−^ n = 4). Open circle denotes raw data for each group. After behavioral test, myelination level was examined by LFB (**F**,**G**) or MBP staining (**H**,**I**). Scale bar: 500 μm. Quantitative analysis of LFB (**G**) or MBP staining (**I**) by optical density in middle corpus callosum (eight sections per mouse in LFB stain and six sections per mouse in MBP stains, region from bregma 0.02 mm to −0.82 mm, WT n = 6, D2R^−/−^ n = 4). Data are presented as mean ± SEM. White bars indicate non-stressed animals and black bars indicate stressed animals. NSt: non-stressed mice, St: stress-imposed mice. ***P < 0.001 versus NSt mice; ^##^P < 0.01, ^###^P < 0.001 versus genotype. ^+++^P < 0.001 versus lithium treatment (Two-way ANOVA post hoc test).

We next examined the expression of D2R in the oligodendrocytes corpus callosum using fluorescent *in situ* hybridization (FISH) with D2R-specific probe (RNAscope *in situ* hybridization). With the same section, we performed the immunohistochemistry with Olig2 antibody to identify D2R expression in the oligodendrocytes. This combined FISH and immunohistochemistry revealed that co-expression of D2R mRNAs (red) and Olig2 proteins (green) in the corpus callosum with approximately 26 percent of Olig2-positive cells found to express D2R (Fig. 6A). As a positive control, same D2R probe was used to perform FISH with brain sections containing caudate putamen (CPu) area where D2R expression is abundant (Fig. 6A, right panel).

We analyzed whether stress exposure or lithium treatment can modulate the expression of D2R in the corpus callosum, using FISH analysis. We observed that D2R mRNA expression was not significantly changed by chronic stress, nor by the lithium treatment in the corpus callosum (Fig. 6B,C).

We next investigated whether the activation of D2R can regulate stress-induced myelin loss, using a selective D2R agonist, quinpirole. WT and D2R^−/−^ mice, which were subjected to chronic stress for 14 days with or without quinpirole treatment, were evaluated with the forced swim test (FST) and further examined for myelination changes (Fig. 6D). Quinpirole treatment decreased the immobility time in stressed WT mice to levels of non-stressed control WT mice (79 ± 21 sec, n = 6 in control WT-NSt mice; 138 ± 10 sec, n = 6 in WT-St mice; 60 ± 28 sec, n = 6, in quinpirole-treated WT-St mice, stress effect, P = 0.0073; quinpirole effect, P = 0.0294, Fig. 6E) in the FST. However, quinpirole treatment had no effect on immobility time of D2R^−/−^ mice (89 ± 23 sec, n = 4 in control D2R^−/−^-NSt mice; 139 ± 9 sec, n = 4 in D2R^−/−^-St mice; 139 ± 13 sec, n = 4, in quinpirole treated D2R^−/−^-St mice; WT × D2R^−/−^ stress effect, P = 0.0073, stress × quinpirole interaction in WT mice: F_1,20_ = 2.046, P = 0.168, Fig. 6E).

Quinpirole treatment effectively prevented the myelin loss in stressed WT mice, as revealed by LFB and MBP staining (Fig. 6F,G). LFB staining within the middle corpus callosum (eight sections, region from bregma 0.02 mm to −0.82 mm, that showed severe myelin loss with LFB and MBP staining, Fig. 1C–F) showed an increase in the intensity of LFB staining in quipirole-treated stressed WT mice, (WT-St mice, n = 6, 46 ± 8% of control WT-NSt mice, n = 6; 96 ± 5%, n = 6 in quinpirole treated WT-St mice, Fig. 6F,G). However, we detected no effect of quinpirole in D2R^−/−^ mice (62 ± 9% of control WT-NSt mice in D2R^−/−^-NSt mice, n = 4; 44 ± 13%, n = 4 in D2R^−/−^-St and 59 ± 17%, n = 4, in quinpirole-treated D2R^−/−^-St mice, stress × genotype interaction: F_1,16_ = 4.751, P = 0.0446, stress × quinpirole interaction in WT mice: F_1,20_ = 12.45, P = 0.0021, Fig. 6F,G). In parallel, quinpirole treatment increased the intensity of MBP staining in stressed WT mice (WT-St mice, n = 6, 57 ± 7% of control WT-NSt mice, n = 6; 90 ± 5%, n = 6 in quinpirole treated WT-St mice, Fig. 6H,I), but not in D2R^−/−^ mice (53 ± 10% of control WT-NSt mice in D2R^−/−^-NSt mice, n = 4; 43 ± 1%, n = 4 in D2R^−/−^-St and 62 ± 14%, n = 4, in quinpirole-treated D2R^−/−^-St mice, stress × genotype interaction: F_1,16_ = 8.955, P = 0.0086, stress × quinpirole interaction in WT mice: F_1,20_ = 20.27, P = 0.0002, Fig. 6H,I). Together, these findings suggested that activation of D2R prevented chronic stress-induced myelin damage, and this effect was not seen in mice lacking D2R.

## Discussion

Increasing evidence suggests that abnormalities in myelin are associated with mental illness, pointing to an important role of oligodendrocytes in this disease^16,41^. However, the underlying molecular mechanisms remain unclear. Anatomical or brain imaging studies of patients with severe mental illnesses, including major depressive disorder (MDD), bipolar disorder (BD), and schizophrenia (SZ) have revealed deficiencies in brain myelination^19,42–46^. Expression profiling studies using post mortem brains of patients with major depressive disorder indicated that depressive disorder might be associated with changes in genes related to myelin, oligodendroglia, and synaptic functions^15,19^. In animal models of depression, reduced glial cell number and morphological changes in OLs are observed^47–49^; these effects are similar to the abnormalities in OLs reported in patients with depression. These alterations in OLs would induce impairment of myelin integrity, which in turn can deteriorate the axon-myelin interactions, and could be key cellular and molecular events implicated in synaptic modification occurring in chronic-stress induced depression. Here, we mainly investigated the corpus callosum, because the corpus callosum is the largest white matter structure in the brain, having high content of myelin. Numerous findings demonstrated that abnormal myelination in the corpus callosum is associated with bipolar disorder^50–54^. Benedetti *et al*., for example, reported that adverse childhood experiences alter white matter microstructure including corpus callosum, in patients with bipolar disorder^55^. As well, brain imaging studies with major depressive disorder (MDD) patients showed that MDD patients have widespread abnormalities in the corpus callosum^56–58^. Therefore, together with other animal studies^59–61^, these observations indicate a strong association between alterations of the myelination of the corpus callosum with chronic stress-induced depressive behaviors.

In the present study, we demonstrated that chronic stress induced-myelin loss was regulated by Wnt/β-catenin signaling, and that this regulation was dependent on the DA system through D2R. Recent findings identified a role of the Wnt/β-catenin signaling in myelination, with initial reports suggesting, rather, an inhibitory role for Wnt signaling in OPC differentiation and OL maturation during early postnatal development^29,31,62^. Other studies using genetic and pharmacological antagonists to inhibit components of Wnt/β-catenin signaling also demonstrated that inhibition promoted OPC differentiation and remyelination in both *in vitro* and *in vivo* models^63–66^. In contrast, numerous reports have shown that activation of Wnt signaling induces myelination. For instance, Tawk *et al*.^32^, have demonstrated a key role for Wnt/β-catenin signaling in the expression of myelin-related genes and myelin sheath compaction in both in the peripheral and central nervous systems using loss-of-function analyses in zebrafish embryos. Inhibition of Wnt/β-catenin signaling resulted in hypomyelination without affecting Schwann cell and OL generation, or axonal integrity. It has also been reported that *in vivo* activation or inhibition of canonical Wnt signaling, respectively, increased or decreased the number of Olig2-positive and PDGFRα-positive cells in the adult subependymal zone, thereby showing the contribution of Wnt/β-catenin pathway to oligodendrogliogenesis, myelin gene expression, and myelinogenesis^67^. Furthermore, it was demonstrated that in *Tcf4 gene* KO mice, there are few changes in the number of OPCs; however, there is severe defects in oligodendrocyte maturation^62,68^. It has been reported that Tcf4 is expressed during remyelination stages in patients with multiple sclerosis and in the cuprizone mouse model of myelin loss, thereby supporting a role for Tcf4 in remyelination^62,68–70^. These contradictory findings indicate that Wnt pathway plays an important role in OL maturation and myelin formation, most likely in a complex manner, which depends on developmental stage and corresponding molecular networks. One possible explanation is that interaction of the Wnt signaling with other pathways can tightly adjust the balance between the negative and positive effects on myelination. In this context, our present finding suggests a possible link between DA and Wnt system through D2R regulation of remyelination, particularly under the stressful stimuli.

Given that lithium, generally known as a glycogen synthase kinase-3β inhibitor, treatment normalized chronic stress-induced Wnt signaling inactivation we also examined whether lithium treatment in the modulation of myelin in corpus callosum would involve glycogen synthase kinase-3β (GSK3β) signaling. However, immunofluorescent histochemistry performed using antibody to GSK3β revealed no signal of GSK3β in the corpus callosum (Supplementary Fig. 1A) while robust labeling of GSK3β could be detected in the cingulate gyrus (cg), caudate putamen (CPu) and primary somatosensory cortex (S1 cortex) (Supplementary Fig. 1B,C). Therefore, based on our data, we believe that the regulation we observed here involves rather a link between dopamine/lithium and Wnt/ β-catenin/Tcf4 signaling without direct involvement of GSK3β in this regulation of myelin in the corpus callosum.

Dopamine systems are considered important modulators of stress-related depressive illness which is associated with altered response to reward, thereby contributing to the pathophysiology of stress-evoked depression^2–4,6,71^. This deficiency in DA transmission involves not only reduced DA release but also abnormal density and sensitivity of dopamine receptors, resulting in dysfunction of DA receptors and disrupted DA transmission in response to external stimuli^72,73^. How dopamine systems with dysfunctional DA receptors contribute to the pathophysiology of depression is not yet known.

It has been reported that the dopamine system can regulate OL functions. For example, D2R expression was observed in interfascicular OLs around the peak of myelination, from P15 to P20, in rat brain^21^ and in differentiated OLs from rat cortical OL cultures^22,74^. In addition, a protective role of D2R against oxidative glutamate toxicity and oxygen-glucose deprivation injury in OLs^22^ has been demonstrated as chronic treatment with the D2R antagonist haloperidol reduced expression of myelin protein genes in mice^75^. Disrupted integrity in myelination observed in the corpus callosum of D2R^−/−^ mice in the present study thus raises the possibility that D2R is a potential regulator of OL development and myelination formation. This is consistent with the observations made in this study, in which quinpirole treatment in stressed WT mice protected stress-induced myelin damage, while no such effect is observed in D2R^−/−^ mice.

Another interesting finding of the present study is that in D2R^−/−^ mice, upon chronic stress, Wnt singling pathway-mediated regulation of myelination was blunted, which supports the hypothesis that DA signaling through D2R, in association with the Wnt pathway, is critical for stress-induced myelin loss. Since stress can affect axon-myelin interaction, myelin formation and remyelination in response to stress must be tightly controlled by neurotransmission. Based on our present findings, we suggest that DA, via D2R, can modulate the Wnt pathway, which in turn controls stress-induced myelin loss, thereby contributing to the regulation of white matter integrity in stress-induced neurological disorders. Our findings may explain the complexity of antidepressant treatment resistance, known to be related a hypodopaminergic state, which can result in lack of effect owing to the absence of DA system activation. Indeed, it has been demonstrated that some D2R agonists such as pramipexole and aripiprazole are effective in patients with depression who have failed to respond to previous medications^9,10,76,77^. Our finding that the effect of lithium treatment was dependent on the presence of D2R may be related to this clinical situation. Consideration of DA signaling would not only be particularly important for synaptic modification in the regulation of myelination associated with the Wnt/β-catenin axis but also be promising as effective antidepressant medication.

## Methods

### Mice

D2R^−/−^ mice (B6.129S2- *Drd2*^*tm1Low*^/J) were obtained from the Induced Mutant Resource at the Jackson Laboratory (Bar Harbor, ME). The D2R^−/−^ mice and WT littermates originated from the breeding of D2R+/− heterozygotes. Mice were maintained in a specific pathogen-free barrier facility under constant conditions of temperature and humidity, and on a 12:12-h light-dark schedule. Animal care and handling were performed in accordance with standards approved by the Institutional Animal Care and Use Committee of Korea University.

### Chronic Immobilization Stress

Behavioral experiments were performed with male D2R^−/−^ and WT control mice at 11–13 weeks of age. Age-matched WT and D2R^−/−^ mice were housed individually and allowed to acclimatize to the cage for 1 week. Mice assigned to stress groups were restrained in a ventilated acrylic restrainer fit to allow the animal to breathe but not to move otherwise. For chronic stress, they were immobilized for 2 h once daily for 14 days. WT and D2R^−/−^ mice were placed in the home cage for restraint stress. Mice assigned to non-stressed groups were untouched in the home cage, whereas their counterparts were subjected to restraint stress.

### Cuprizone-Induced Myelin loss

7-week-old WT male mice were fed 0.2% (w/w) cuprizone (bis-cyclohexanone oxaldihydrazone, 14690, Sigma-Aldrich, St. Louis, MI) mixed into a regular diet of ground standard chow (Purina Certified Rodent Diet, USA). The cuprizone diet was maintained for 5 weeks. Cuprizone ingestion results in a reproducible pattern of corpus callosum myelin loss over this 5-week period. Control “non-treated” mice were maintained on normal chow pellets.

### Lithium treatment

Mice received either a lithium-supplemented diet (0.2% LiCO_3_; TD.08019, Teklad, Harlan) or the control diet. Since chronic lithium treatment can result in polyuria and increased excretion of sodium^78^, all mice were provided a second drinking bottle containing 0.9% saline to counteract potential ion imbalance^79^.

### Quinpirole treatment

(−)-Quinpirole hydrochloride (Tocris, Bristol, UK) was dissolved in 0.9% saline and was administered intraperitoneally once daily (one hour before restraint) at doses of 5 mg/kg/day during chronic stress. Control group received an equivalent volume of 0.9% saline.

### Tissue preparation

Animals were anesthetized by intraperitoneal (i.p.) injection of 1.6 μl of Zoletil and 0.05 μl of xylazine (Rompun, Bayer) per gram of body weight and transcardially perfused with 4% paraformaldehyde in PBS. Brains were removed and postfixed 4 h at 4 °C. The brains were cryoprotected in 30% sucrose-PBS solution for 2 days. Brains were then frozen and 20 µm thick consecutive coronal sections were obtained on a Cryotome (Leica CM 1900, Germany).

### Luxol fast blue stain

Six sections were prepared from specific regions within the middle corpus callosum that showed severe myelin loss in LFB stain (region from bregma 0.14 mm to −0.82 mm, 0.16-mm intervals). Brain sections were dehydrated with ethanol grades, incubated in 0.1% luxol fast blue stain solution (solvent blue 38 0.1 g, ethyl alcohol 95% 100 ml, glacial acetic acid 0.5 ml) at 56 °C overnight, and washed with distilled water. Sections were dipped in 0.05% lithium carbonate 30 sec and 70% regent alcohol for gray and white matter differentiation. The slices were incubated in cresyl violet stain for 1 min, dipped in 70% regent alcohol, dehydrated through 2 changes of absolute alcohol, and mounted with Permount (Fisher Scientific). The myelin loss area and integrated optical density (IOD) were analyzed with MetaMorph 6.1 software (Universal Imaging Corp.; Downingtown, PA).

### Electron microscope histological analysis (TEM)

After anesthesia, mice were perfused with 4% paraformaldehyde and 2.5% glutaraldehyde in 0.1 M phosphate buffer. Dissected tissues containing the corpus callosum (bregma −0.46 mm) were prepared from 1 mm parasagittal serial brain sections. The tissues were buffered washed, fixed in 1% osmium tetroxide, dehydrated in graded ethanol series, transitioned in propylene oxide, and embedded in TAAB resin (TAAB Laboratories). Semi-thin sections (1 µm mid-sagittal, lateral 0 mm) were cut, mounted on glass slides, and stained in toluidine blue for general assessment in the light microscope. Subsequently, 50~60-nm ultra-thin sections were cut with a diamond knife (Diatome), mounted on copper slot grids coated with parlodion, and stained with uranyl acetate and lead citrate for examination on an electron microscope (Hitachi; H7650). Ten random images within each field were collected at 12000× magnification. The digitalized images were then analyzed on a MetaMorph image analysis system (Universal Imaging Corp.).

### G-ratio analysis

Digitized, non-overlapping electron micrographs of corpus callosum were analyzed for axon diameter and g-ratio. Images were analyzed using MetaMorph 6.1 software. The g-ratio measurements were obtained by manually drawing lines across 2 perpendicular diameters for both the axons and for the axons plus myelin sheath. At least 300 axons per brain were measured. The analysis was modified such that the inner diameter of compact myelin (instead of the axon diameter) was divided by the outer diameter of the myelin sheath (because the inner tongue was frequently enlarged in demyelinated animals, but not in controls). Axons with diameters typical of unmyelinated fibers (<0.3 mm) were excluded from analysis. Fibers with prominent outfoldings in the plane of section were also excluded.

### MBP Immunohistochemistry

Six sections were prepared within the middle corpus callosum that showed severe myelin loss in LFB stain (region from bregma 0.14 mm to −0.82 mm, 0.16-mm intervals). The 20-µm free-floating sections were washed in 0.1 M PBS (pH 7.4) three times for 10 min and placed in 0.1 M PBS solution containing 0.3% H_2_O_2_ and 50% methanol for 30 min. Sections were incubated in blocking solution (0.3% Triton X-100, 1% BSA in 0.1 M PBS) for 30 min and then in primary antibody a-MBP (1:2000; Abcam, 7349) at 4 °C overnight. This was followed by incubations at room temperature in biotinylated secondary antibody (anti-rat IgG 1:1000; Vector Laboratories, Burlingame, CA) for 30 min and in an ABC kit (Vector Laboratories, Burlingame, CA) for 30 min. Sections were reacted with DAB (Vector Laboratories, Burlingame, CA). The 0.1 M PBS rinses were performed between each step. Sections were mounted, dehydrated, and cover slipped.

### Double-labeled immunofluorescence

Three sections were prepared from the middle corpus callosum that showed severe myelin loss in LFB stain (region from bregma −0.22 mm, −0.46 mm and −0.70 mm). The 20 µm free-floating sections were pre-treated with 1% NaOH and 1% H_2_O_2_ for 20 min, incubated in 0.3% glycine in PBS for 10 min, and blocked with a blocking solution (3% bovine serum albumin, and 0.3% Triton X-100 in PBS, pH 7.4) for 1 h at room temperature. Tissue sections were incubated overnight at 4 °C with a-TCF4 (1:500; Santacruz, sc-13027), a-APC(CC1) (1:250; Millipore, OP80), a-Olig2 (1:250; Santacruz, sc-19969), a-Olig2 (1:350; Millipore, AB9610), ABC (Anti-Active-β-Catenin Antibody clone 8E7; 1:100; Millipore, 05–665), and PDGFRα (1:200; rat 558774, BD Biosciences). After rinsing in PBS, the double-stained sections were incubated at room temperature for 1 h with Alexa Fluor 488 anti-Rabbit (donkey IgG) (1:500; Life Technologies, A-21206), Alexa Fluor 568 anti-mouse (donkey IgG) (1:1000; Life Technologies, A-10037), and Alexa Fluor 488 anti-Goat (donkey IgG) (1:500; Life Technologies, A-11057). After rinsing in PBS, the sections were mounted in Vectashield (Vector Laboratories) to prevent fading of the immunofluorescence stain. Sections were examined on a confocal laser scanning system, (LSM 700, Zeiss, Berlin, Germany).

### Fluorescent *in situ* hybridization (FISH) and Olig2 immunofluorescence-histochemistry

Animals were anesthetized and transcardially perfused with 4% paraformaldehyde in PBS. Brains were removed and postfixed 4 h at 4 °C. Dehydrate the tissue in 10%, 20%, 30% sucrose in 1X PBS at 4 °C until the tissue sinks to the bottom of the container. Tissue were frozen at −20 °C in the Optimal Cutting Temperature (OCT) embedding media and 14 μm-thick coronal sections were placed on SuperFrost® Plus slides (Fisher Scientific #12-550-15) and air dry the slides for 20 min at −20 °C. FISH was performed according to RNAscope® ISH Tissue instructions (Advanced Cell Diagnostics (ACD), ACD 320850, Hayward, CA) for fixed-frozen tissue. Slides were digested with protease (1:15, diluted in PBS) for 30 min at 40 °C. Sections were incubated for 2 h at 40 °C in a hybridization oven (HybEZ™, ACD) with RNAscope® Probe- mm-Drd2-C3 (ACD 406501-C3) probes to detect mouse D2R mRNA. Ppib probe (ACD 313911-C3), a mouse housekeeping gene, was used as a positive control probe, and a bacterial dapB probe (ACD 310043-C3) was used as a negative control. Sections were then incubated with Amp 1- FL, Amp 2- FL, Amp 3- FL, and 4-FL–Alt A solutions according to instructions. Wash with 0.1 M PBS twice, blocked with a blocking solution (5% bovine serum albumin, and 0.2% Triton X-100 in PBS, pH 7.4) for 1 h at room temperature. Tissue sections were incubated overnight at 4 °C with a-Olig2 (1:500; Millipore, AB9610). After rinsing in PBS, the double-stained sections were incubated at room temperature for 1 h with Alexa Fluor 488 anti-Rabbit (donkey IgG) (1:1000; Life Technologies, A-21206). After counterstaining with DAPI for 30 sec, slides were cover-slipped with Vectashield (Vector Laboratories) to prevent fading. Slides were imaged using confocal laser scanning system, (LSM 700, Zeiss, Berlin, Germany). Probe channel C3 was observed with a filter for Atto 647. To count D2R expressing cells in Olig2-positive cells in WT mice (Fig. 6A), cells were counted in the corpus callosum region within the range from bregma −0. 22 mm to −0.70 mm (4 sections per mouse) and summed data were used for quantification. Approximately 400 cells expressing D2R or Olig2 were counted and only cells with a D2R mRNA puncta fluorescent signals or Olig2 fluorescent signals that filled the cell body were included for analysis. Individual puncta were counted using MetaMorph image analysis system (Universal Imaging Corp.). In a similar way, the expression levels of D2R mRNA transcripts in stress-imposed WT mice with and without lithium treatment (Fig. 6C) was analyzed, puncta were counted in the corpus callosum region within the region from bregma −0.22 mm, −0.46 mm and −0.70 mm (3 sections per mouse) and summed data were used for quantification.

### Forced swim test

Mice were transferred to the experimental room 60 min before the onset of the experiment to allow for habituation and reduce stress (brightness of the experimental room was 70 lux). The forced swim test was performed by dropping a mouse in water and recording its behavior. The apparatus consisted of a plastic cylinder (internal diameter, 10 cm; height, 25 cm) containing a 19 cm column of water maintained at 22 ± 2 °C. The mouse was thus not able to support itself by touching the bottom of the apparatus with its paws or tail. An animal was considered to be immobile when it floated in an upright position and made only small movements to maintain its head above water. The total duration of immobility, which is thought to reflect a depressive state, was determined during a 6-min test session.

### Statistical analysis

Data are presented as means ± s.e.m. and were analyzed with one-way or two-way analysis of variance (ANOVA) followed by Bonferroni’s post hoc test. A P-value of < 0.05 was considered statistically significant.

## Electronic supplementary material

Supplementary Information
