# Publisher Correction: Role of Dopamine D2 Receptor in Stress-Induced Myelin Loss

**DOI:** 10.1038/s41598-018-22954-x

**Published:** 2018-04-11

**Authors:** Mi-Hyun Choi, Ji Eun Na, Ye Ran Yoon, Hyo Jin Lee, Sehyoun Yoon, Im Joo Rhyu, Ja-Hyun Baik

**Affiliations:** 1 Molecular Neurobiology Laboratory, Department of Life Sciences, Korea University, Seoul, 02841 Korea; 2 Department of Anatomy, College of Medicine, Korea University, Seoul, 02841 Korea

Correction to: *Scientific Reports* 10.1038/s41598-017-10173-9, published online 14 September 2017

This Article contains a repeated typographical error in the legends of Figures 1, 2 and 3 where

“D2R-St”

should read:

“D2R^−/−^-St”

