# Supplementary material for: Role of Dopamine D2 Receptor in Stress-Induced Myelin Loss
Source: Sci Rep. 2017 Sep 14;7:11654. doi: 10.1038/s41598-017-10173-9 (PMC5599541; doi:10.1038/s41598-017-10173-9)
Supplement: Supplementary file 1 — Supplementary Information [file 41598_2017_10173_MOESM1_ESM.pdf]

Role of Dopamine D2 Receptor in Stress-Induced Myelin Loss

Mi-Hyun Choi[1], Ji Eun Na[2], Ye Ran Yoon[1], Hyo Jin Lee[1], Sehyoun Yoon[1], Im Joo Rhyu[2] and Ja-Hyun Baik[1*]

[1]Molecular Neurobiology Laboratory, Department of Life Science, Korea University, Seoul 02841, Korea, [2]Department of Anatomy, College of Medicine, Korea University, Seoul 02841, Korea.

*To whom correspondence should be addressed:

Dr. Ja-Hyun Baik, Molecular Neurobiology Laboratory, Department of Life Science, Korea University, Seoul 02841, Korea. Tel: 82-2-3290-3455, Fax: 82-2-927-9028. E-mail: jahyunb@korea.ac.kr

Supplementary Information

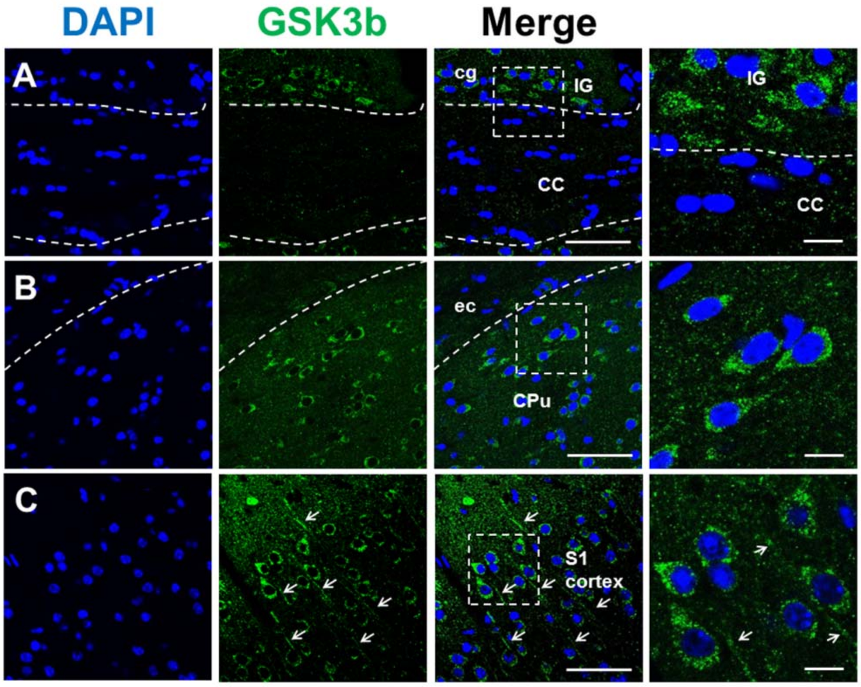

Supplementary Figure 1. Glycogen synthase kinase-3beta (GSK3β) expression in adult mouse brain. Immunofluorescent histochemistry performed using GSK3β antibody to examine GSK3β expressing cells in adult mouse brain. **(A-C)** GSK3β immunostaining displays robust labeling in the cingulate gyrus (cg), indusium griseum (IG), caudate putamen (CPu) and primary somatosensory cortex (S1 cortex), while there is no staining in the corpus callosum (CC) and external capsule (ec). **(C)** Neuronal cytoplasm and the segment of the apical dendrites is strongly immunolabeled in S1 cortex (arrows). White dashed box indicate magnified image. Scale bars: 50 μm in low magnification images and 10 μm in high magnification images.

Methods

Tissue preparation Animals were anesthetized by intraperitoneal injection of 1.6 μl of Zoletil and 0.05 μl of xylazine (Rompun, Bayer) per gram of body weight and transcardially perfused with 4% paraformaldehyde in PBS. Brains were removed and postfixed 4 h at 4℃. The brains were cryoprotected in 30% sucrose-PBS solution for 2 days. Brains were then frozen and 10 μm thick consecutive coronal sections on gelatin coated slide were obtained on a Cryotome (Leica CM 1900, Germany).

Glycogen synthase kinase-3beta (GSK3β) immunofluorescent histochemistry The 10 μm sections were pre-treated with 10 mM citrate buffer (pH6.0) at 95°C for 10 min, and blocked with a blocking solution (3% bovine serum albumin, and 0.3% Triton X-100 in PBS, pH 7.4) for 1 h at room temperature. Tissue sections were incubated overnight at 4°C with a-GSK3β (1:100; Cell Signaling, #9832). After rinsing in PBST (0.3% triton X-100 in PBS), the double-stained sections were incubated at room temperature for 1 h with Alexa Fluor 488 anti-mouse (donkey IgG) (1:200; Life Technologies, A-150105). After rinsing in PBST, counterstain with DAPI and the sections were mounted in Vectashield (Vector Laboratories) to prevent fading of the immunofluorescence stain. Sections were examined on a confocal laser scanning system, (LSM 700, Zeiss, Berlin, Germany).
